# Psychometric validation of a cognition and social participation bolt-on for the EQ-5D-5L in SARS-CoV-2 infected German healthcare workers

**DOI:** 10.1007/s11136-026-04226-8

**Published:** 2026-04-01

**Authors:** Ines Buchholz, Laura Lüdtke, Martin Härter, M. F. Bas Janssen

**Affiliations:** 1 Department of Medical Psychology, Institute for Psychotherapy, University Medical Center Hamburg-Eppendorf, Hamburg, Germany; 2 Maths in Health, Klimmen, The Netherlands

**Keywords:** Quality of life, Healthcare workers, EQ-5D-5L, Psychometrics, Cognition, Social participation, Germany

## Abstract

**Purpose:**

COVID-19 can result in long-term impairments, including cognitive difficulties and restrictions in social participation, which may not be fully captured by EQ-5D-5L. This study examined whether adding cognition (CO) and social participation (SP) bolt-ons improves EQ-5D-5L’s measurement properties in German healthcare workers (HCW) with SARS-CoV-2 infection.

**Methods:**

N = 3335 HCW with self-reported occupational COVID-19 completed an online survey including EQ-5D-5L, two candidate bolt-ons (CO, SP), and validated self-report instruments (e.g., Post-COVID Syndrome PCS-Score, PHQ-4, PTSD screening, SSD-12, WAI, WHODAS). Psychometric analyses covered distributional characteristics (response pattern, missing values, ceiling), construct (convergent and divergent) validity, known-groups validity, and explanatory power.

**Results:**

Both bolt-ons showed acceptable distributional properties; adding CO modestly reduced overall ceiling effect, while adding SP resulted in negligible change (‘11111’ = 18.8% vs. ‘111111’_CO_ = 14.8% and ‘111111’_SP_ = 17.8%). Construct validity was supported by expected correlation patterns, i.e. r_CO,PCS-Score_ = 0.63, r_SP, WHODAS_ = 0.71. Known-groups validity improved with the inclusion of bolt-ons, as reflected by higher or comparable relative efficiency (RE) compared with EQ-5D-5L in most group comparisons for CO (11/13, RE = 0.94–1.22), whereas this was observed in approximately half of group comparisons for SP (6/13, RE = 0.89–1.18). In multivariate models, adding CO to the EQ-5D-5L resulted in a small to moderate increase in explained variance for PCS symptom severity (Δ adj. R^2^_CO_ = 0.04–0.07), whereas adding SP had a negligible impact (Δ adj. R^2^_SP_ ≤ 0.01).

**Conclusion:**

While CO improved ceiling, construct, and known-groups validity of the EQ-5D-5L in SARS-CoV-2 infected HCW, the added value of SP appeared limited.

**Supplementary Information:**

The online version contains supplementary material available at 10.1007/s11136-026-04226-8.

## Abstract (plain language summary)

- Many people who have had COVID-19 continue to experience problems with thinking, memory, and social participation. These issues are common, but current tools used to measure health – like the EQ-5D-5 L – do not fully capture them. This study looks at whether adding new items (called “bolt-ons”) about cognition (CO) and social participation (SP) can help improve how we measure health of individuals with post-COVID syndromes.
- We studied a large group of healthcare workers who reported post-COVID syndromes after being infected with SARS-CoV-2 at work. We tested how well the new items on CO and SP worked when added to the EQ-5D-5 L.
- Our results suggest that adding CO improved validity, reduced ceiling effects, and enhanced the EQ-5D-5 L’s explanatory power, particularly for post-COVID symptom burden. While SP improved known-groups validity in some comparisons, it added little explanatory power, suggesting a weaker overall contribution in our sample of individuals with post-COVID syndromes.
- The findings support the targeted use of bolt-ons to improve measurement precision in COVID and studies examining post-infection recovery, including physical, cognitive, and social outcomes. Including a CO bolt-on to the EQ-5D-5L may better inform clinical assessments and health policy decisions related to long COVID care and rehabilitation planning, while the SP bolt-on showed limited added value.

**Supplementary Information:**

The online version contains supplementary material available at 10.1007/s11136-026-04226-8.

## Introduction

The EQ-5D-5L is a widely used instrument for measuring health-related quality of life (HRQoL). It assesses five domains on a five-level severity-scale, providing a standardized and simple approach to compare health status across populations and diseases [1]. Its brevity and generic nature have contributed to its extensive use in health technology assessment and outcome research.

Despite these strengths, the EQ-5D-5L may incompletely capture condition-specific aspects of health, such as cognitive and psychosocial functioning [2–10]. To address these limitations, supplementary "bolt-on" dimensions have been proposed and increasingly studied, targeting health aspects not covered by the core domains [11–17].

The COVID-19 pandemic has highlighted the relevance of such extensions. Many individuals experience ongoing symptoms after acute infection, referred to as Long COVID (4–12 weeks) or Post-COVID-Syndrome (PCS, > 12 weeks) [18, 19], including symptoms like fatigue, cognitive difficulties, respiratory problems, and sequelae liked reduced social and occupational functioning [20–23]. Cognitive problems—such as memory issues, attention deficits, and slowed processing—are prominent among persons with PCS and can negatively affect mental health, work performance, and social participation [24, 25]. Emerging longitudinal evidence from the Norwegian adult population indicate that individuals who had COVID-19 reported poorer social participation on PROMIS-29 compared with pre-pandemic levels, exceeding minimal important change thresholds. Interestingly, respondents whose family or partner had COVID-19 also reported poorer outcomes in anxiety and social participation, suggesting broader social and relational impacts of the pandemic [25]. About 9 months into the pandemic, EQ-5D-5L and PROMIS-29 scores showed slightly poorer overall health compared with 1 year earlier, highlighting that even in general population samples social participation may be particularly affected. These findings underscore the relevance of including social participation alongside cognition as bolt-ons to capture the broader functional impact of PCS.

During the COVID-19 pandemic, the EQ-5D-5L was extensively used to monitor population health, evaluate health inequalities, and generate utility values for economic evaluations [20, 25–33]. However, most studies focused on general population samples or non-COVID-specific cohorts. A systematic review and meta-analysis of nearly 200 EQ-5D studies demonstrated a substantial reduction in HRQoL among COVID-19 patients, with pain/discomfort and anxiety/depression as the most affected domains [34]. Risk factors for lower HRQoL included older or younger age, female gender, disease severity, comorbidities, and post-COVID symptoms [25, 34].

Longitudinal evidence from the CORona Follow-Up study shows that a substantial proportion of former COVID-19 patients reported persistent symptoms 24 months post-infection, accompanied by reduced HRQoL, as reflected by lower EQ-5D-5L, EQ VAS and health utility scores compared with non-infected controls [20]. Janols et al. (2024) demonstrated in Swedish post-COVID patients that the EQ-5D-5L partly captures fatigue and memory/concentration problems but poorly reflects dyspnea, and that adding symptom-specific bolt-ons enhances the explained variance of overall HRQoL [35].

Healthcare workers (HCW) are a particularly relevant group. High occupational exposure, sustained workload, and psychosocial stress during the pandemic increase the risk of SARS-CoV-2 and post-COVID sequelae [36, 37]. In Germany, approximately 6.1 million individuals—around 13% of the workforce—are employed in the healthcare sector [38]. Alongside high infection rates [39], European studies report persistent psychological, cognitive and functional burden among HCW during and after the pandemic, including elevated levels of depression, anxiety, stress, and reduced work functioning [40–43]. In the context of post-COVID conditions, these sustained demands may translate into persistent cognitive impairments and restrictions in social participation with direct implications for daily functioning and work ability, and direct relevance for HRQoL assessment. These characteristics make HCW a key population for evaluating whether extended HRQoL instruments better capture post-COVID–related health deficits.

Against this background, this study aimed to evaluate the psychometric properties of the EQ-5D-5L with and without a cognition and a social participation bolt-on in a randomly selected cohort of German HCW who acquired SARS-CoV-2 infection at work.

## Methods

### Data collection and study population

This secondary data analysis used cross-sectional data from an online survey (REDCap) funded by the Federal Ministry of Education and Research (funding code: 01EP2110A). The original study aimed to examine the prevalence of COVID-19 symptoms and Post-COVID Syndrome (PCS) in German HCW. More information about the study design can be found in the study protocol [44, 45].

The initial study sample included 20,000 HCW in Germany with confirmed SARS-CoV-2 infection during occupational exposure, randomly selected from a population of approximately 120,000 HCW by the employer’s liability insurance association (German statutory accident insurance provider for non-state institutions within the health and welfare service sectors, Berufsgenossenschaft für Gesundheitsdienst und Wohlfahrtspflege, BGW). Inclusion criteria required participants to be HCW in regular contact with patients, insured by the BGW, with a confirmed SARS-CoV-2 infection (positive PCR test) before February 14, 2023. The present analyses are based exclusively on data collected at baseline from subjects with a confirmed SARS-CoV-2-infection (positive PCR-Test).

### Outcome measures

Data was collected using validated German self-report measures (for the entire survey instrument please see [45]). Questionnaires were presented in a fixed order. For sample characterization, sociodemographic information, such as age, sex, marital status, education, employment, occupation, course and intensity of symptoms were assessed. This study used the data collected with the following questionnaires:

### Brief resilience scale

The Brief Resilience Scale (BRS) is a validated 6-item self-report measure designed to assess an individual's ability to recover from stress. Items are rated on a 5-point Likert scale, with higher scores indicating greater resilience.

### EQ-5D-5L and bolt-ons

The EQ-5D-5L includes a descriptive system comprising five health dimensions (mobility (MO), self-care (SC), usual activities (UA), pain/discomfort (PD), anxiety/depression (AD)) each rated on a 5-point severity scale ranging from *no problems* (1) to *extreme problems/unable to* (5), and a vertical visual analogue scale (EQ VAS), on which respondents rate their overall health from 0 (*the worst health you can imagine*) to 100 (*the best health you can imagine*). Responses from the five dimensions can be combined into a 5-digit health string, where ‘11111’ represents the best possible health profile, and ‘55555’ represents the worst.

The EQ-5D-5L was administered last, followed by two bolt-ons—cognition (CO) and social participation (SP)—and the EQ VAS.

The bolt-ons are not formally part of the EQ-5D descriptive system; their use and translation from English into German were approved by EuroQol Research Foundation. The English and German item wordings are provided in Supplementary Material 1.

Bolt-ons were selected using a symptom-guided, literature-based approach in line with current EQ-5D bolt-on recommendations [11].

Cognition was included because cognitive problems are among the most common long-term sequelae of COVID-19 [20] and seem to be insufficiently captured by the core EQ-5D-5L [35]. It is also the earliest [10] and most frequently tested bolt-on in empirical EQ-5D research, with applications targeting diverse item formulations (e.g., concentration, memory, and thinking ability) across diverse populations [16].

SP was selected to capture pandemic-related social and occupational limitations, which have been shown to affect HRQoL, and to be associated with psychological distress and fatigue [46]. Among the various social domain bolt-ons described in the literature [6, 7, 17, 47, 48], this item focuses on participation in social life rather than interpersonal relationships alone [30], aligning with functional impairments commonly reported after COVID-19.

Both bolt-ons were previously applied in the POPCORN study, one of the first studies employing EQ-5D bolt-ons in a COVID-19 context [30]. They had a single-item format with a recall period of 3 months; the response format followed the structure of the EQ-5D-5L descriptive system. As the term ‘cognition’ is more used in a scientific context than in everyday German language, examples and explanations from the POPCORN study [30] and Finch et al. (2021) were provided in parenthesis following the dimension title to enhance comprehension and completion [11].

To avoid redundancy and respondent burden, no additional EQ-5D-5L bolt-ons addressing other key post-COVID symptoms such as fatigue or respiratory problems were included, as these were assessed using the Post-COVID Syndrome (PCS) severity score.

### PCS – severity of post COVID syndrome

The Post-COVID Syndrome PCS-Score is a symptom-based severity classification tool designed to quantify the extent of long-term symptoms following COVID-19 infection [49]. It comprises 12 symptom complexes, including fatigue, neurological issues, sleep disturbances, and musculoskeletal pain, among others. Each symptom complex is assessed through binary items and weighted (2–7 points) based on clinical relevance. As respondents first indicate the presence of a symptom (no/yes, corresponding to EQ-5D-5L level 1 vs. ≥ 2), followed by severity levels that are conceptually aligned with EQ-5D-5L levels 2–5, the items can be considered “bolt-on-like” measures tailored to the post-COVID population.

### German version of the moral injury symptom and support scale for health professionals (G-MISS-HP)

The G-MISS-HP is a German version of the MISS-HP, measuring moral injury and related impairment in healthcare professionals, capturing distress from perceived moral value violations at work with 11 items on a 10-point Likert scale [50, 51].

### PHQ-4 – anxiety and depression

The Patient Health Questionnaire-4 (PHQ-4) is a brief screening tool consisting of four items to assess symptoms of depression and anxiety [52]. Each symptom is rated on a 4-point frequency scale (*0* = *not at all, 1* = *several days, 2* = *more than half the days, 3* = *nearly every day*). The total score ranges from 0 to 12, with a score of 5 or higher indicating the potential presence of clinical anxiety or depression [53].

### SSD-12 – symptom-related thoughts, feelings, and behaviours

The Somatic Symptom Disorder–B Criteria Scale (SSD-12) [54–56] is a 12 item self-report instrument based on DSM-5 criteria, assessing cognitive, emotional, and behavioural aspects of somatic symptom disorder on a 5-point frequency scale from 0 (“*never*”) to 4 (“*very often*”), yielding a total score from 0 to 48.

### Short screening scale for DSM IV posttraumatic stress disorder

The Short Screening Scale for DSM-IV Posttraumatic Stress Disorder (PTSD) is a brief diagnostic tool designed to identify individuals with PTSD based on DSM-IV criteria, with scores ≥ 4 indicating probable PTSD [57].

### WHODAS 2.0

The WHO Disability Assessment Schedule 2.0 (WHODAS 2.0), assesses functioning and disability over the past 30 days across six domains using a 5-point Likert scale [58]. Based on the International Classification of Functioning, Disability and Health (ICF), it enables cross-cultural comparisons of health and disability. This study used the 12-item short version.

### Work ability index (WAI)

The Work Ability Index is a 24-item self-assessment tool designed to evaluate a person’s capacity to meet current and future work demands across seven dimensions [59, 60]: current work ability compared with lifetime best, work ability in relation to job demands, number of diseases (assessed using a 14-category disease list indicating physician- or self-diagnosed conditions and whether they existed prior to the first SARS-CoV-2 infection), work impairment due to diseases, sickness absence during the past year, own prognosis of work ability in 2 years, and mental resources. It is widely used to identify risks for reduced work ability and to guide preventive measures in occupational health.

### Statistical analysis

We followed the analytical framework used in previous studies to test the distributional properties, convergent and divergent validity, known-groups validity, and explanatory power of bolt-ons [12]. Psychometric properties were first assessed for the EQ-5D-5L, followed by the EQ-5D-5L with added bolt-on(s). Socio-demographic, occupational, and health-related characteristics of the baseline population are presented. All analyses were performed using Stata 18.5.

### Distributional properties

For each EQ-5D-5L item and bolt-on, we analysed the number and proportion of missing values (feasibility) and the response distribution across the five levels to confirm relevance and endorsement of all levels. Stratified analyses were also conducted by PCS severity. Missing values were not imputed for subsequent analyses. To ensure that the EQ-5D-5L items and the bolt-ons were able to detect differences at the upper end of the scale, we examined (i) the proportion of respondents reporting *"no problems"* (level 1) on each EQ-5D-5L dimension and bolt-on item separately (item-level ceiling), and (ii) the proportion reporting *"no problems"* across all dimensions of the EQ-5D-5L (‘11111’) and the EQ-5D-5L + each bolt-on item (‘111111’) combined (profile-level ceiling). The frequency of persons reporting “*no problems*” on each EQ-5D-5L item but any problems on the bolt-on was examined using cross-tabulations.

### Convergent and divergent validity

To investigate construct validity, Spearman’s correlation coefficients were calculated between bolt-on items and the EQ-5D-5L items, the EQ VAS, and relevant scores or items of other measures, including BRS, G-MISS-HP, PCS-Score, PHQ-4, Short Screening Scale for PTSD, SSD-12, WAI and WHODAS. For scores and items targeting related constructs (convergent) such as EQ-5D-5L dimension anxiety/depression and PHQ-4, we expected at least moderate correlations (r = 0.40–0.59); for those who measure unrelated constructs (divergent validity) such as EQ-5D-5L dimension mobility and PHQ-4 weak (r=0.2– < 0.4) to no correlations according to available guidelines [61].

### Discriminative (known groups) validity

Known-groups validity was examined using an anchor-based approach, comparing mean level sum scores (LSS; transformed to a 0–100 scale [62]) for the EQ-5D-5L alone and with bolt-ons across externally defined subgroups based on clinical and other indicators (e.g., physician-diagnosed post-COVID condition, number of sick leave days, PHQ-4). Clinically meaningful groups were defined using established cut-offs from the literature. Symptoms of anxiety and depression were categorized using the PHQ-4 [52] as normal (0–2), mild (3–5), moderate (6–8), and severe (9–12). Somatic symptom burden was assessed with the SSD-12 [63–65]; in the face of lacking recommended thresholds, previously applied cut-offs derived from primary care and population-based studies were used (SSD-12 < 13 vs. ≥ 13). These cut-offs were analyzed both alone and in combination with PHQ-4 severity (PHQ-4 < 8 and SSD-12 < 13 vs. PHQ-4 ≥ 8 and SSD-12 ≥ 13). Post-COVID symptom severity was defined using the PCS-Score [49] as no (0 points), mild (0.01–10.75), moderate (10.76–26.25), and severe (> 26.25). A positive screen for post-traumatic stress disorder was defined as a score ≥ 4 on the short Screening Scale for DSM IV PTSD [57]. Work ability, assessed with the WAI, was categorized as excellent (44–49), good (37–43), moderate (28–36), or low (7–27) [66].

We hypothesized that adding bolt-ons would improve the EQ-5D-5L's ability to distinguish between groups expected to differ on relevant constructs. Known-group validity was evaluated using relative efficiency (RE), defined as the ratio of F-statistics for multi-group comparisons or squared t-statistics for binary comparisons, with the EQ-5D-5L without bolt-ons as the reference. Statistical significance of changes in discriminatory ability was assessed using 95% bootstrap confidence intervals based on 3000 replications, following [67].

### Explanatory power

Linear regression models were used to assess the explanatory power of EQ-5D-5L dimensions with and without bolt-ons in explaining variance in the severity of self-reported post-COVID symptoms with the baseline PCS-Score and the baseline EQ VAS score as dependent variables and the EQ-5D-5L and bolt-on items as predictors.

Explanatory power refers to how well the EQ-5D-5L dimensions and bolt-ons account for variability in the respective outcome measure in a multivariable context. Analyses were run using the total sample and subsamples of the most prevalent health conditions, defined as more than n = 200 cases self-reporting a diagnosis in WAI.

The same modelling approach was applied using EQ VAS, PHQ-4, SSD-12, and WAI as dependent variables in the total sample to investigate whether EQ-5D-5L dimensions with and without bolt-ons capture constructs beyond overall self-rated health as measured by EQ VAS.

Univariable models were estimated for descriptive purposes only. The primary analyses focused on multivariable models including all EQ-5D-5L dimensions, with adjusted R^2^ and incremental R^2^ (Δ adjusted R^2^) used to quantify the additional explanatory value of bolt-ons beyond the core EQ-5D-5L.

## Results

### Study population

A total of n = 3335 individuals (mean age: 50.8 years; 85.6% female) completed the assessment (Table 1). About one-third (33.3%) of participants were in the age group of 50–59 years. Most participants were care staff/nurses (73.3%), with 44.6% working full-time and 46% part-time. The majority had no migration experience (87.4%), 40% held a German high school or university of applied sciences entrance qualification, and 58.9% reported two or more infections.

**Table 1:** Characteristics of the patient population

Variables^1^	n	%
Total sample	3335	100.0
Female sex	2852	85.6
Age (years), mean ± sd (min–max)	50.8 ± 11.4 (19–83)	
20–29	192	5.8
30–39	430	12.9
40–49	706	21.3
50–59	1114	33.6
60 +	876	26.4
Occupation (professional group)	3333	
Care staff/nurse	2442	73.3
Physician	315	9.5
Therapeutical staff/therapist	203	6.1
Advisor	20	0.6
Other	353	10.6
Current working situation	2969	
Full-time employed	1323	44.6
Part-time employed	1365	46.0
Working in marginal employment/on an hourly basis	56	1.9
In reintegration	34	1.2
Unemployed	72	2.4
Retired	119	4.0
Living situation	2969	
Alone	474	16.0
With others	2495	84.0
Migration experience	2971	
No	2607	87.4
Yes, I myself (1^rst^ generation)	276	9.3
Yes, at least one parent has migration experience	99	3.3
Highest level of education	3000	
Higher education entrance qualification	1198	40.0
German intermediate school certificate	1426	47.5
Other	376	12.5
Size of residence	2.959	
< 1.000 inhabitants	430	14.5
Up to 10.000	963	32.5
Up to 35.000	613	20.7
Up to 100.000	396	13.4
> 100.000	557	18.8
Number of infections	3331	
1	1368	41.1
2	1581	47.5
≥ 3	382	11.5
EQ VAS (0–100), mean ± sd	n = 2934, 71.1 ± 20.0	

^1^n (number of cases) and % (percent), otherwise reported; sd, standard deviation

### Feasibility and distribution properties

Table 2 presents the response distribution and proportion of missing values for the EQ-5D-5L dimensions and bolt-on items. Respondents without any item responses (n = 234) were excluded from analyses, assuming survey break-off. Among the remaining respondents, missing values ranged from 0.4% to 0.6% for the EQ-5D-5L dimensions and bolt-ons. Missing values for the EQ VAS were approximately 5% (n = 167). Regarding the response distribution, the proportion of respondents reporting *no problems* ranged from 27.4% for PD to 92.7% for SC. The proportion reporting *severe* or *extreme problems* was generally low (< 10%), with the highest rates observed for CO (9.3%), followed by PD (8.6%).

**Table 2:** Feasibility and distribution of EQ-5D-5L and bolt-on item responses in the total sample

	Missing values^1^		No problems		Slight problems		Moderate problems		Severe problems		Extreme problems/unable to		Full health^2^	
	n	%	n	%	n	%	n	%	n	%	n	%	n	%
EQ-5D items														
Mobility	12	0.4	1.718	55.6	712	23.1	477	15.4	176	5.7	6	0.2	571	18.8
Self-care	12	0.4	2.862	92.7	151	4.9	52	1.7	16	0.5	8	0.3		
Usual activities	14	0.4	1.640	53.1	834	27.0	434	14.0	161	5.2	18	0.6		
Pain/discomfort	20	0.6	845	27.4	845	37.7	811	26.3	230	7.5	33	1.1		
Anxiety/depression	18	0.5	1519	49.3	1519	29.1	488	15.8	153	5.0	25	0.8		
Bolt-ons														
Cognition	19	0.6	1088	35.3	1170	37.9	538	17.5	234	7.6	52	1.7	450	14.8
Social participation	17	0.5	1780	57.7	694	22.5	381	12.4	185	6.0	44	1.4	540	17.8
EQ VAS	167	5.0	–	–	–	–	–	–	–	–	–	–	50	1.7

^1^Individuals who did not answer any of the EQ-5D-5L items (n = 234) were excluded from missingness analysis

^2^EQ-5D-5L (‘11111’) (+ bolt-on) or EQ VAS = 100, respectively

**Fig. 1:**
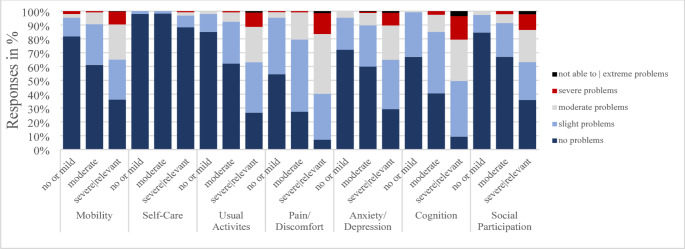
Distribution of responses based on self-rated PCS severity (PCS-Score T_1_): no or mild (0–10.75), moderate (10.76–26.25), severe (> 26.25) PCS

Among respondents reporting *no problems* with MO, 14.8% reported *moderate* to *extreme* cognitive problems (Table 3). Similarly, 23.4% of those without self-care limitations reported cognitive difficulties, and 16.3% reported at least moderate problems with SP. Stratified analysis by symptom severity revealed the added value of both bolt-ons, which becomes especially apparent with increasing severity of post-COVID symptomatology (Fig. 1).

**Table 3:** Number and proportion of patients reporting “*no problems*” on the EQ-5D-5L dimension by bolt-ons (T_1_)

Bolt-on		Number and proportion of patients reporting “*no problems*” with…					
		Mobility	Self-care	Usual activities	Pain/discomfort	Anxiety/depression	’11111’
Cognition	No problems	835 (48.9)	1065 (37.5)	920 (56.4)	533 (63.2)	856 (56.5)	450 (78.8)
	Slight problems	621 (36.3)	1116 (39.2)	583 (35.7)	237 (28.1)	492 (32.5)	111 (19.4)
	Moderate problems	188 (11.0)	458 (16.1)	110 (6.7)	60 (7.1)	121 (8.0)	9 (1.6)
	Severe problems	58 (3.4)	175 (6.2)	16 (1.0)	12 (1.4)	41 (2.7)	1 (0.0)
	Extreme problems	7 (0.4)	30 (1.1)	2 (0.1)	2 (0.1)	4 (0.3)	0 (0.0)
	Total^1^	1709 (100)	2844 (100)	1631 (100)	844 (100)	1514 (100)	571 (100)
Social participation	No problems	1252 (73.2)	1746 (61.4)	1352 (82.9)	684 (81.3)	1265 (83.6)	540 (94.9)
	Slight problems	293 (17.1)	637 (22.4)	221 (13.6)	108 (12.8)	172 (11.4)	26 (4.6)
	Moderate problems	118 (6.9)	328 (11.5)	45 (2.8)	35 (4.2)	52 (3.4)	2 (0.4)
	Severe problems	39 (2.3)	119 (4.2)	13 (0.8)	10 (1.2)	22 (1.5)	1 (0.2)
	Extreme problems	8 (0.5)	16 (0.6)	0 (0.0)	4 (0.5)	3 (0.2)	0 (0.0)
	Total^1^	170 (100)	2846 (100)	1631 (100)	841 (100)	1514 (100)	569 (100)

^1^Corresponds to ceiling in EQ-5D-5L items

#### Convergent and divergent validity

CO and SP show moderate to high correlations with all EQ-5D-5L dimensions (e.g., r_CO,UA_ = 0.63, r_SP,UA_ = −0.65, r_SP,PD_ = −0.63) except SC (r_CO,SC_ = 0.27, r_SP,SC_ = 0.33). Except for moral injury (G-MISS-HP) and resilience (BRS), which show weak correlations of r < 0.27, correlations with other measures are generally moderate to high, particularly with broader constructs of functioning, (dis)ability and health (WHODAS: r_CO,WHODAS_ = 0.69, r_SP,WHODAS_ = 0.71, WAI: r_CO,WAI_ = −0.63, r_SP,WAI_ = −0.60, EQ VAS: r_CO,VAS_ = r_SP,VAS_ = −0.58, Table 4).

**Table 4:** Spearman's rank correlation coefficients; pairwise; T_1_: EQ-5D-5L items, bolt-on items and other measures

Variables*	MO	SC	UA	PD	AD	CO	SP
EQ-5D-5L items							
MO							
SC	0.354						
UA	0.570	0.381					
PD	0.603	0.316	0.587				
AD	0.377	0.266	0.507	0.451			
Bolt-ons							
CO	0.405	0.271	0.587	0.478	0.513		
SP	0.425	0.329	0.626	0.445	0.586	0.552	
EQ VAS	− 0.541	− 0.319	− 0.654	− 0.633	− 0.542	− 0.583	− 0.575
Other measures							
SSD-12	0.484	0.280	0.601	0.566	0.583	0.574	0.554
PHQ-4	0.422	0.249	0.547	0.482	0.727	0.561	0.574
PTSD	0.412	0.266	0.555	0.470	0.581	0.562	0.589
BRS	− *0.029*	*0.006*	− *0.002*	− *0.002*	0.114	*0.027*	*0.037*
G-MISS-HP	0.170	0.104	0.211	0.171	0.272	0.214	0.207
WAI	− 0.538	− 0.289	− 0.693	− 0.641	− 0.573	− 0.632	− 0.604
WHODAS	0.614	0.366	0.749	0.628	0.633	0.687	0.706
PCS total score	0.514	0.287	0.603	0.609	0.498	0.631	0.508
PCS items^1^							
1 Smell or taste disorders	0.201	0.103	0.201	0.208	0.156	0.231	0.160
2 Fatigue (chronic exhaustion, tiredness)	0.463	0.268	0.575	0.530	0.484	0.573	0.479
3 Lack of physical resilience (e.g., full capacity not regained, shortness of breath)	0.494	0.268	0.588	0.538	0.426	0.540	0.449
4 Joint or muscle pain	0.503	0.260	0.470	0.625	0.364	0.409	0.356
5 Complaints in the throat, nose or ear area (e.g., hoarseness, pain or scatchiness)	0.251	0.146	0.286	0.306	0.245	0.289	0.247
6 Lung or breathing difficulties (e.g., coughing, whistling or wheezing)	0.348	0.211	0.377	0.344	0.283	0.330	0.290
7 Heart complaints (e.g., extrasystoles, palpitations, chest pain)	0.293	0.194	0.338	0.348	0.311	0.350	0.295
8 Gastrointestinal complaints (e.g., abdominal pain, diarrhea, vomiting, nausea)	0.284	0.233	0.314	0.318	0.275	0.294	0.318
9 Complaints or abnormalities of the nervous system or memory (e.g., concentration problems)	0.395	0.222	0.545	0.491	0.471	0.734	0.469
10 Skin complaints (e.g., hair loss, rash, itching)	0.196	0.111	0.239	0.250	0.213	0.236	0.203
11 Signs of infection (e.g., chills, fever, flu-like feeling)	0.262	0.203	0.261	0.243	0.216	0.253	0.226
12 Sleep disorders (e.g., difficulties falling asleep or sleeping through the night)	0.398	0.242	0.469	0.472	0.443	0.474	0.404
13 Other health restrictions	0.270	0.188	0.302	0.282	0.216	0.249	0.242
14 Sexual complaints (e.g., loss of libido, erectile dysfunction, pain during sexual)	0.240	0.163	0.302	0.255	0.265	0.309	0.295

All *p* < 0.01 except those in italic letters. *n_min_ = 1819, n_avg_ = 2758, n_max_ = 3153. ^1^n_min_ = 2804, n_avg_ = 3100, n_max_ = 3301

G-MISS-HP, Moral Injury Symptom and Support scale for Health Professionals, German version; PCS-Score, Post-COVID syndrome PCS-Score; PHQ-4, Patient Health Questionnaire; SSD-12, Somatic Symptom Disorder-B criteria scale; PTSD, Short screening scale for DSM-IV posttraumatic stress disorder; WAI, Work Ability Index; WHODAS, WHO Disability Assessment Schedule 2.0

EQ-5D-5L items display convergent validity, with moderate to high correlations with corresponding measures, such as anxiety/depression (r_AD,PHQ-4_ = 0.73) and posttraumatic stress (r_AD,PTSD_ = 0.58). Conversely, correlations with instruments measuring different constructs are weak (e.g., r_BRS_ = −0.03–0.04, r_G-MISS-HP_ = 0.10–0.27), demonstrating the divergent validity of the EQ-5D-5L.

SC stands out as the only dimension with minimal correlation to other instruments, suggesting that it either captures a unique aspect of health or exhibits limited variance, which may reduce the power to estimate its associations properly. Excluding SC, correlations with the EQ VAS are moderate to strong, ranging from r = −0.54 (MO, AD) to r = −0.65 (UA), reinforcing the validity of the EQ-5D-5L in assessing overall health.

### Known-groups validity

Inclusion of CO significantly improved discrimination in 10 of 13 comparisons compared to the EQ-5D-5L alone, with relative efficiencies (REs) ranging from 1.10 (95% CI 1.06–1.14) to 1.22 (95% CI 1.16–1.28, Table 5). EQ-5D-5L + SP improved discrimination in 6 of 13 comparisons, with REs of 1.06 (95% CI 1.01–1.10) to 1.18 (95% CI 1.13–1.24).

As expected, neither bolt-on improved RE for PCS severity prior to infection. Adding CO significantly improved discrimination of PCS severity groups at baseline and during the most severe COVID-19 episode compared with the EQ-5D-5L alone, whereas EQ-5D-5L + SP performed similarly to the standard EQ-5D-5L.

For work outcomes, results were mixed: EQ-5D-5L + SP showed better discrimination of current sick leave status due to post-COVID symptoms (RE_SP_ 1.18, 95% CI 1.13–1.24 vs. RE_CO_ = 1.11, 95% CI 1.05–1.16). In contrast, EQ-5D-5L + CO performed better for WAI-assessed work ability and for mental health measures (PHQ-4, SSD-12), while no additional benefit was observed when these measures were combined (Table 5).

**Table 5:** Known groups validity of the EQ-5D-5L (+ bolt-ons): baseline mean LSS ± SD with RE (ref: EQ-5D-5L) with Bootstrap SE and 95% CI

Known groups	n	%	EQ-5D-5L	EQ-5D-5L + CO	EQ-5D-5L + SP
PCS diagnosed by physician^1^					
Yes	765	23.3	71.5 ± 17.9	68.9 ± 17.9	70.9 ± 18.7
No	2159	76.7	85.8 ± 14.1	84.8 ± 14.1	85.9 ± 18.7
RE ± SE (95% CI)		Ref	1.22 ± 0.03 (1.16–1.28)	1.06 ± 0.02 (1.01 1.10)	
Full days absent from work due to post-COVID symptoms					
0 days	216	7.1	90.5 ± 11.3	89.2 ± 11.5	90.5 ± 11.4
< 10	220	7.2	90.5 ± 12.3	89.9 ± 12.1	91.0 ± 11.8
10–24	1167	38.2	86.1 ± 14.2	85.1 ± 14.3	86.3 ± 14.4
25–99	1185	38.7	80.1 ± 15.5	78.7 ± 15.4	80.3 ± 15.6
≥ 100 days	271	8.9	67.0 ± 18.6	64.3 ± 18.5	65.5 ± 19.4
RE ± SE (95% CI)		Ref	1.17 ± 0.03 (1.11–1.22)	1.10 ± 0.25 (1.05–1.15)	
Current incapacity for work due to COVID-illness^2^					
Yes	212	6.4	58.2 ± 17.4	55.4 ± 17.2	55.7 ± 17.6
No	3095	93.6	84.2 ± 14.8	82.9 ± 14.9	84.3 ± 14.9
RE ± SE (95% CI)		Ref	1.11 ± 0.03 (1.05–1.16)	1.18 ± 0.03 (1.13–1.24)	
Work ability according to WAI					
Very good (44–49)	265	10.9	97.5 ± 4.0	97.2 ± 4.1	97.7 ± 3.6
Good (37–43)	703	29.0	92.8 ± 7.9	91.9 ± 7.8	93.2 ± 7.3
Moderate (28–36)	830	34.3	82.9 ± 11.2	81.4 ± 10.9	83.1 ± 11.1
Poor (7–27)	625	25.8	66.3 ± 15.3	64.1 ± 14.8	65.5 ± 15.8
RE ± SE (95% CI)		Ref	1.18 ± 0.02 (1.13–1.22)	1.08 ± 0.02 (1.04–1.11)	
PHQ-4					
Normal (0–2)	1525	50.2	90.6 ± 10.8	89.6 ± 11.1	90.9 ± 10.6
Mild (3–5)	985	32.4	78.3 ± 13.7	76.7 ± 13.3	78.4 ± 13.6
Moderate (6–8)	340	11.2	67.3 ± 15.1	65.1 ± 14.7	66.2 ± 15.4
Severe (9–12)	186	6.1	55.7 ± 16.9	53.7 ± 16.2	53.6 ± 17.2
RE ± SE (95% CI)		Ref	1.12 ± 0.02 (1.08–1.16)	1.03 ± 0.02 (0.99–1.06)	
SSD-12					
No impairment (< 13)	1182	41.4	91.4 ± 9.4	90.5 ± 9.6	91.8 ± 9.1
Impairment (≥ 13)	1671	58.6	73.3 ± 16.3	71.4 ± 16.1	72.9 ± 16.9
RE ± SE (95% CI)		Ref	1.12 ± 0.02 (1.08–1.16)	1.03 ± 0.02 (0.99–1.06)	
Combination of PHQ-4 and SSD-12					
SSD-12 < 13	1182	35.4	91.4 ± 9.4	90.5 ± 9.6	91.8 ± 9.1
SSD-12 ≥ 13 AND PHQ-4 < 8	1637	49.1	79.3 ± 15.1	77.8 ± 15.1	79.4 ± 15.3
SSD-12 ≥ 13 AND PHQ-4 ≥ 8	516	15.5	70.7 ± 21.7	68.9 ± 21.9	69.4 ± 22.6
RE ± SE (95% CI)		Ref	1.10 ± 0.02 (1.06–1.14)	1.13 ± 0.02 (1.09–1.17)	
Before first infection					
No (0)	1321	43.9	85.8 ± 15.7	84.4 ± 16.8	85.6 ± 16.3
Mild (> 0 and ≤ 10.75)	839	27.9	81.9 ± 15.5	80.4 ± 15.7	81.9 ± 15.9
Moderate (> 10.75- ≤ 26.25)	627	20.8	79.4 ± 15.9	78.3 ± 15.9	79.5 ± 16.1
Severe/relevant (> 26.25)	222	7.4	74.2 ± 17.3	72.9 ± 16.8	74.3 ± 17.7
RE ± SE (95% CI)		Ref	0.94 ± 0.04 (0.86–1.01)	0.89 ± 0.03 (0.82–0.95)	
Baseline (T_1_)					
No or mild (0 to ≤ 10.75)	865	28.5	94.7 ± 7.4	94.4 ± 7.1	94.9 ± 7.1
Moderate (> 10.75 to ≤ 26.25)	687	22.6	88.0 ± 10.3	86.9 ± 10.0	88.2 ± 10.5
Severe/relevant (> 26.25)	1482	48.9	72.7 ± 16.5	70.7 ± 16.1	72.5 ± 16.9
RE ± SE (95% CI)		Ref	1.21 ± 0.02 (1.17–1.24)	0.99 ± 0.01 (0.97–1.02)	
During most severe infection					
No or mild (0 to ≤ 10.75)	214	7.1	95.4 ± 7.4	95.5 ± 6.9	95.8 ± 6.9
Moderate (> 10.75- ≤ 26.25)	500	16.6	91.3 ± 10.3	90.7 ± 10.2	91.4 ± 10.5
Severe/relevant (> 26.25)	2301	76.3	79.1 ± 16.6	77.4 ± 16.6	79.0 16.9
RE ± SE (95% CI)		Ref	1.21 ± 0.02 (1.16–1.25)	1.00 ± 0.02 (0.97–1.03)	
Short screening scale for DSM-IV PTSD					
No PTSD (< 4)	1618	53.9	90.3 ± 10.6	89.5 ± 10.7	90.8 ± 10.3
PTSD (≥ 4)	1381	46.1	73.4 ± 16.7	71.5 ± 16.4	72.8 ± 17.1
RE ± SE (95% CI)		Ref	1.16 ± 0.02 (1.11–1.19)	1.13 ± 0.02 (1.09–1.16)	
Number of pre-existing conditions^1^					
0	1107	34.1	86.7 ± 15.7	85.4 ± 15.9	86.5 ± 16.0
1	673	20.7	84.8 ± 15.4	83.4 ± 15.8	84.9 ± 15.6
2	528	16.3	82.4 ± 15.4	80.8 ± 15.8	82.4 ± 15.8
3	450	13.9	80.3 ± 14.6	78.9 ± 14.8	80.5 ± 15.1
4 +	488	15.0	74.7 ± 16.7	73.5 ± 16.4	74.8 ± 17.3
RE ± SE (95% CI)		Ref	0.96 ± 0.02 (0.89–1.02)	0.91 ± 0.03 (0.85–0.97)	
Number of physician-diagnosed diseases^1^					
0	1040	32.9	87.9 ± 14.9	86.8 ± 15.0	87.9 ± 15.1
1	575	18.2	87.3 ± 13.2	86.1 ± 13.4	87.4 ± 13.4
2	524	16.6	83.8 ± 14.3	82.3 ± 14.7	83.8 ± 14.8
3–4	684	21.7	79.5 ± 14.9	77.9 ± 15.1	79.6 ± 15.2
5 +	335	10.6	68.4 ± 17.3	66.8 ± 17.1	68.2 ± 17.9
RE ± SE (95% CI)		Ref	1.03 ± 0.02 (0.98–1.07)	0.96 ± 0.02 (0.92–1.01)	

^1^Self-reported; ^2^Currently on sick leave due to the consequences of COVID-19 illness; CI, Confidence Interval; CO, Cognition bolt-on; LSS, Level Sum Scores; PHQ-4, Patient Health Questionnaire-4; PTSD, Post-Traumatic Stress Disorder; RE, Relative Efficacy; ref, Reference; SD, Standard Deviation; SE, Standard Error; SP, Social participation bolt-on; SSD-12, Somatic Symptom Disorder-B criteria scale

#### Explanatory power

Across all outcomes, multivariate models showed that adding a single bolt-on improved the explanatory power of the EQ-5D-5L, with the largest incremental gains observed for PCS (Δ adj. R^2^ = 0.05) and WHODAS (Δ adj. R^2^ = 0.03); univariate regressions revealed domain-specific patterns (Table 6).

**Table 6:** The explanatory power of EQ-5D-5L and bolt-ons for EQ VAS, SSD-12, PHQ-4, PCS-Score, PTSD, WHODAS and WAI (all T_1_)

Dimensions	EQ VAS (0–100)		SSD-12 (0–48)		PHQ-4 (0–12)		PCS-Score (0–59)		PTSD (0–24)		WHODAS (12–60)		WAI (7–49)	
	Adj. R^2^	Δ	Adj. R^2^	Δ	Adj. R^2^	Δ	Adj. R^2^	Δ	Adj. R^2^	Δ	Adj. R^2^	Δ	Adj. R^2^	Δ
Individual dimensions														
Mobility	0.343		0.253		0.191		0.268		0.181		0.424		0.313	
Self-care	0.156		0.095		0.088		0.088		0.117		0.239		0.116	
Usual activities	**0.479**		**0.368**		0.287		0.363		0.297		**0.583**		**0.506**	
Pain/discomfort	0.411		0.330		0.228		0.372		0.223		0.418		0.425	
Anxiety/depression	0.317		0.358		**0.550**		0.250		0.370		0.413		0.332	
Cognition (CO)	0.357		0.330		0.301		**0.397**		0.296		0.484		0.404	
Social participation (SP)	0.369		0.329		0.353		0.264		**0.398**		**0.567**		0.401	
Combinations														
EQ-5D-5L	0.593		0.524		0.588		0.495		0.459		0.725		0.627	
EQ-5D-5L + CO	**0.611**	0.018	**0.548**	0.024	**0.605**	0.017	**0.558**	0.053	0.482	0.023	0.759	0.034	**0.655**	0.028
EQ-5D-5L + SP	0.601	0.008	0.535	0.011	0.601	0.013	0.501	0.006	**0.506**	0.047	**0.770**	0.045	0.645	0.018

Δ, incremental R^2^ compared to EQ-5D-5L; EQ VAS, Visual Analog Scale of the EQ-5D-5L; PCS-Score, Post-COVID syndrome PCS-Score; PHQ-4, Patient Health Questionnaire; SSD-12, Somatic Symptom Disorder-B criteria scale; PTSD, Short screening scale for DSM-IV posttraumatic stress disorder; WAI, Work Ability Index; WHODAS, WHO Disability Assessment Schedule 2.0; Δ, incremental R^2^ compared to EQ-5D-5L

In multivariable models, predicting PCS symptom severity across prevalent health conditions (Table 7), adding CO to the EQ-5D-5L consistently resulted in a notable increase in explanatory power (Δ adj. R^2^ = 0.04–0.06), while adding SP yielded negligible changes in model performance (adj. R^2^ ≤ 0.01). Univariate models showed the strongest crude associations for CO in most (4/6) conditions. Only for psychological and hormonal/metabolic conditions other items performed better.

**Table 7:** The explanatory power (adjusted R^2^) of the EQ-5D-5L and bolt-on items (baseline PCS-Score as dependent variable) for the 6 most prevalent health conditions (n > 200)

PCS-Score (0–59)														
Dimensions	Total sample		Musculoskeletal disorders		Cardiovascular diseases		Psychological impairments		Hormonal/metabolic diseases		Respiratory diseases		Neurological and sensory disorders	
	Adj. R^2^	Δ	Adj. R^2^	Δ	Adj. R^2^	Δ	Adj. R^2^	Δ	Adj. R^2^	Δ	Adj. R^2^	Δ	Adj. R^2^	Δ
Individual dimensions														
Mobility	0.268		0.233		0.238		0.189		0.268		0.212		0.257	
Self-care	0.088		0.085		0.085		0.070		0.099		0.064		0.090	
Usual activities	0.363		0.300		0.323		0.217		**0.351**		0.277		0.326	
Pain|discomfort	0.372		0.284		0.321		**0.251**		0.331		0.282		0.281	
Anxiety|depression	0.249		0.227		0.209		0.117		0.241		0.223		0.237	
Bolt-ons														
Cognition (CO)	**0.397**		**0.333**		**0.339**		0.216		0.337		**0.346**		**0.329**	
Social participation (SP)	0.263		0.224		0.216		0.138		0.225		0.219		0.271	
EQ-5D-5L(+ bolt-ons)														
EQ-5D-5L	0.495		0.427		0.44		0.33		0.462		0.403		0.451	
EQ-5D-5L + CO	**0.558**	0.063	**0.487**	0.060	**0.498**	0.058	**0.383**	0.053	**0.511**	0.049	**0.477**	0.074	**0.490**	0.039
EQ-5D-5L + SP	0.501	0.006	0.431	0.004	0.442	0.002	0.335	0.005	0.461	− 0.001	0.405	0.002	0.456	0.005

Δ, incremental R^2^ compared to EQ-5D-5L; PCS-Score, Post-COVID syndrome PCS-Score

When predicting HRQoL using the EQ VAS (Table 8), multivariable models indicated a modest additional contribution of CO (Δ adj. R^2^ = 0.01–0.02), while adding SP to the EQ-5D-5L resulted in negligible changes (Δ adj. R^2^ ≤ 0.01).

**Table 8:** The explanatory power (adjusted R^2^) of the EQ-5D-5L and bolt-on items (baseline EQ VAS Score as dependent variable) for the 6 most prevalent health conditions (n > 200)

EQ VAS (0–100)														
Dimensions	Total sample		Musculoskeletal disorders		Cardiovascular diseases		Psychological impairments		Hormonal/metabolic diseases		Respiratory diseases		Neurological and sensory disorders	
	Adj. R^2^	Δ	Adj. R^2^	Δ	Adj. R^2^	Δ	Adj. R^2^	Δ	Adj. R^2^	Δ	Adj. R^2^	Δ	Adj. R^2^	Δ
Individual dimensions														
Mobility	0.343		0.304		0.321		0.294		0.377		0.351		0.333	
Self-care	0.156		0.162		0.148		0.161		0.177		0.166		0.207	
Usual activities	**0.479**		**0.462**		**0.479**		**0.387**		**0.490**		**0.426**		**0.452**	
Pain/discomfort	0.411		0.386		0.381		0.307		0.406		0.377		0.373	
Anxiet/depression	0.317		0.299		0.286		0.222		0.313		0.275		0.262	
Bolt-ons														
Cognition (CO)	0.357		0.325		0.308		0.243		0.298		0.298		0.334	
Social participation (SP)	0.369		0.338		0.360		0.276		0.333		0.338		0.362	
EQ-5D-5L (+ bolt-ons)														
EQ-5D-5L	0.593		0.576		0.578		0.515		0.603		0.564		0.568	
EQ-5D-5L + CO	**0.611**	0.018	**0.594**	0.018	**0.592**	0.014	**0.529**	0.014	**0.617**	0.014	**0.584**	0.020	**0.583**	0.015
EQ-5D-5L + SP	0.601	0.008	0.584	0.008	0.589	0.011	0.521	0.006	0.607	0.004	0.575	0.011	0.573	0.005

Δ, incremental R^2^ compared to EQ-5D-5L

## Discussion

This study investigated whether adding two existing bolt-ons, cognition and social participation, improved the measurement properties of the EQ-5D-5L in a German cohort of HCW with confirmed occupational SARS-CoV-2 infection and with prominent post-COVID syndromes. It is one of the first to evaluate the added value of CO and SP bolt-ons to the EQ-5D-5L in a large, well-defined post-COVID sample. The use of multiple validated instruments and a 12-month follow-up allowed for robust assessment of psychometric properties. In addition, known groups and explanatory power analyses were conducted across a wide range of clinically relevant subgroups, enhancing the interpretability and applicability of results.

Both bolt-ons showed acceptable distributions, with a slight reduction in ceiling effects compared to the EQ-5D-5L alone, more pronounced for CO than SP (‘11111’ = 18.8% vs. ‘111111’_CO_ = 14.8% and ‘111111’_SP_ = 17.8%). While most dimensions exhibited high ceilings, CO and pain/discomfort captured a wider spread of reported problems. Construct validity was supported by expected correlation patterns, that is moderate to high correlations with related constructs, e.g. SP and WHODAS 2.0, and no to poor correlations with non-related constructs, e.g. CO and BRS. The inclusion of bolt-ons resulted in comparable or improved known-groups validity across most comparisons, with CO outperforming SP. CO showed the highest explanatory power for PCS-Scores (adj. R^2^ = 0.33–0.39) in univariate models. In multivariate models, the bolt-ons had small to moderate explanatory value.

The addition of a CO bolt-on to the EQ-5D-5L has been explored in various studies to enhance the instrument’s ability to capture cognitive health aspects, particularly in populations with neurological conditions or cognitive impairments. The findings of this study align with and expand upon previous evidence on EQ-5D-5L bolt-ons. Consistent with earlier findings, CO demonstrated added value in terms of construct and known-groups validity [67]. This supports prior work showing that cognitive limitations are not adequately captured by the core instrument [10, 13], especially in populations with neurological or post-infectious conditions [10, 16, 35, 68]. Notably, the ceiling was reduced by the inclusion of bolt-ons, mirroring findings from studies in traumatic brain injury, post-traumatic stress disorder and coeliac disease populations [69–71]. The particularly strong correlation of both CO and SP with generic instruments (e.g. WHODAS 2.0, WAI) underscores their relevance in capturing broader health dimensions.

Recent longitudinal evidence from non-hospitalized post-COVID-19 patients further contextualizes these findings. In a validation study of EQ-5D-5L breathing and cognition bolt-ons, Stavem and Garratt (2026) demonstrated acceptable construct validity for both bolt-ons; however, only the respiratory bolt-on contributed meaningfully to additional variance explained in EQ VAS scores, whereas the cognition bolt-on added little incremental explanatory value [72, 73]. This is consistent with our results, suggesting that cognition bolt-ons primarily enhance content validity, discrimination between clinically relevant groups, and explanatory power for symptom-based outcomes (e.g., PCS severity), rather than substantially improving the explanation of global self-rated health.

To the best of our knowledge, evidence on the SP bolt-on remains limited, as most studies addressing the social aspects of quality of life have focused on social relationships or isolation rather than participation in societal roles and activities [5, 48, 67, 74, 75]. Herein, SP showed weaker performance overall, adding little explanatory value and only small improvements in some known-groups comparisons. This may partly reflect conceptual overlap with the UA dimension, as indicated by the moderate to strong correlations between these dimensions, suggesting that the usefulness of the SP bolt-on may be context-dependent and warranting further validation [17].

### Limitations

Several limitations must be acknowledged. First, the sample consisted exclusively of HCW, which may limit generalizability to the broader post-COVID population. Second, self-reported data are subject to recall and reporting biases, particularly given the retrospective design of the survey. Moreover, self-completion of the cognition bolt-on may be challenging for individuals with cognitive impairment, potentially requiring interviewer or proxy administration [76, 77]; consequently, EuroQol has called for further research on its validity and usability in these populations [78]. Third, interpretation of SSD-12 scores is complicated by varying recommended cut-offs across settings: while a threshold of ≥ 13 seems appropriate for primary care and population-based samples, clinical studies suggest higher thresholds (e.g., ≥ 22 or ≥ 29), highlighting the need for further diagnostic validation [63–65]. Fourth, the absence of objective clinical endpoints—such as medically confirmed rather than self-reported diagnoses/syndromes—limits the strength of conclusions regarding group comparisons based on these anchors. Fifth, although our findings suggest good feasibility of the descriptive system and acceptable feasibility of the EQ VAS, they should be interpreted with caution, as a subset of n = 234 respondents did not complete any EQ-5D-5L items, likely reflecting survey fatigue at the end of a lengthy questionnaire. Sixth, since fatigue and respiratory symptoms were already captured by the PCS-Score, no additional EQ-5D bolt-ons were included; however, evaluating such bolt-ons could further advance the evidence base and merits future research. Finally, the bolt-on items referred to a 3-month recall period, whereas the EQ-5D items referred to ‘*today’,* which may complicate comparisons and affect reported levels. Evidence from SF-12/SF-36 and SF-6D research suggests that differing recall periods can influence mean scores and sensitivity to recent changes, though psychometric structure is generally preserved [79, 80]. Chua et al. (2025) found that EQ-5D-5L variants with longer recall periods (1–4 weeks) showed reduced ceiling effects and superior or comparable reliability, validity, and responsiveness relative to the standard ‘*today*’ version in patients with obstructive airway diseases [81].

Differing recall periods may have introduced recall bias because the nature of the reported construct may affect recall accuracy in different ways. Evidence suggests that longer recall periods can alter the level and variability of self‑reported outcomes in patient‑reported instruments, as recall accuracy appears to deteriorate when respondents are asked to aggregate experiences over extended periods rather than report momentary states [82]. This may be particularly relevant for SP, which reflects variable and context‑dependent activities over time and may therefore be more susceptible to recall and aggregation effects when longer reference periods are used. By contrast, cognitive difficulties are often experienced and internalized as more persistent impairments with less moment‑to‑moment fluctuation, and may therefore be less affected by differences in recall period [83, 84]. Caution is therefore warranted when interpreting differences across dimensions and recall periods.

Against this background, our findings may offer preliminary input for the ongoing development of the EQ-5D Bolt-on Toolbox: the consistent added value of the CO bolt-on aligns with cognition being considered as a candidate domain, whereas the more limited and context-dependent contribution of SP observed here—together with evidence of conceptual overlap and recall sensitivity—may help inform future prioritization and validation efforts.

## Conclusion

Adding a cognition (CO) bolt-on appears to improve specific psychometric properties of the EQ-5D-5L, including distributional properties, known groups validity, and explanatory power, in HCW with self-reported occupational SARS-CoV-2 infection and post-COVID syndromes, whereas a social participation (SP) bolt-on shows some improvement in known-groups validity but adds little explanatory power. Results suggest that particularly CO can improve the instrument’s ability to explain post-COVID health burden/PCS symptom severity, supporting their added value in post-acute care and recovery research. However, their added value in explaining self-reported health outcomes appears limited.

## Supplementary Information

Below is the link to the electronic supplementary material.

Supplementary Material 1
