# Supplementary material for: Psychometric validation of a cognition and social participation bolt-on for the EQ-5D-5L in SARS-CoV-2 infected German healthcare workers
Source: Qual Life Res. 2026 Apr 1;35(5):117. doi: 10.1007/s11136-026-04226-8 (PMC13043573; doi:10.1007/s11136-026-04226-8)
Supplement: Supplementary file 1 — Supplementary Material 1 [file 11136_2026_4226_MOESM1_ESM.docx]

**Appendix 1**. Wording of the EQ-5D-5L bolt-ons used in this study

| **Dimension heading** | **Cognition**, (e. g., memory, concentration, comprehension, thinking, e.g., inability to remember things or difficulty remembering things, forgetting things, slowed thinking) | **Social participation**, doing things with others (e. g., in work, education, leisure, church, cultural and community activities) |
| --- | --- | --- |
| Level 1 | I have *no* problems with cognition | I have *no* problems with social participation |
| Level 2 | I have *slight* problems with cognition | I have *slight* problems with social participation |
| Level 3 | I have *moderate* problems with cognition | I have *moderate* problems with social participation |
| Level 4 | I have *severe* problems with cognition | I have *severe* problems with social participation |
| Level 5 | I have *extreme* problems with cognition | I have *extreme* problems with social participation |

***© EuroQol Research Foundation. EQ-5D^TM^ is a trade mark of the EuroQol Research Foundation. This is a modified EQ-5D reproduced by permission of EuroQol Research Foundation. Reproduction of this EuroQol instrument is not allowed. For use of this EuroQol instrument or any other EuroQol instrument, please submit a request by using the online registration page on*** [***www.euroqol.org***](http://www.euroqol.org)***.***
